# Predictors of mpox infectious periods: findings from a fine and gray sub-distribution hazard model using Vietnamese national mpox data

**DOI:** 10.1017/S0950268825100356

**Published:** 2025-08-04

**Authors:** Thi Ngoc Anh Hoang, Van Ngoc Hoang, Thi Thu Trang Dinh, Ngoc Long Vu, Ha Linh Quach

**Affiliations:** 1 Faculty of Public Health, PHENIKAA University, Hanoi, Vietnam; 2 Viet Nam Administration of Disease Prevention, Ministry of Health, Hanoi, Vietnam; 3 Prevention Research Collaboration, Faculty of Medicine and Health, The University of Sydney, School of Public Health, Sydney, NSW, Australia

**Keywords:** competing risk, fine and gray model, infectious period, Mpox, Vietnam

## Abstract

Investigating risk factors for mpox’s infectious period is vital for preventing this emerging disease, yet evidence remains scarce. This study aimed to identify risk factors associated with the duration of mpox infectiousness among mpox cases in Vietnam. The primary outcome was the duration of the mpox infectiousness, defined between symptom onset and the first negative test result for the mpox virus. Fine and Gray’s regression models were employed to assess the associations between the infectious period and several risk factors while accounting for competing risks of death by mpox. Most mpox cases recovered within 30 days. Patients with HIV or treated at multiple facilities for mpox had lower incidence rates of cleared infection compared to those who were HIV-negative or treated at a single facility. In regression models, patients with mpox symptoms of rash or mucosal lesions (sub-distribution hazard ratios = 0.62, 95% confidence interval = 0.46–0.83), ulcers (0.57, 0.41–0.80), or fever (0.62, 0.46–0.83) had significantly prolonged infectious periods than those without such symptoms. Our findings provided insights for managing mpox cases, especially those vulnerable to prolonged infectious periods in settings with sporadic cases reported.

## Introduction

The global outbreak of mpox since May 2022 is unprecedented, with a significant increase in cases spreading worldwide. On 14 August 2024, the World Health Organization declared the mpox outbreak a public health emergency of international concern, following reports of over 100,000 cases across 122 countries [1]. As an emerging infectious disease, the mpox virus can be transmitted via close contact with rashes, blisters, or sores on the skin, bodily fluids, or contaminated objects of infected persons [2].

The infectious period of mpox, defined as the time during which an individual can transmit the virus to others [3], begins at the onset of symptoms and ends when no new skin lesions appear and existing lesions have crusted [4]. Current literature shows that most mpox cases recover within four weeks; however, complications such as encephalitis, myocarditis, and pneumonia may occur [5, 6]. Clinical symptoms during the mpox infectious period include rash, fever, headache, swollen lymph nodes, fatigue, itchiness, ulcers, sore throat, chills, neck pain, cough, pink eye, nausea, and vomiting [4, 7].

Understanding risk factors associated with prolonged mpox infectious periods is critical for guiding mpox case management and treatment [8, 9]. However, current literature primarily examined risk factors of mpox severity, with most evidence clustered in Africa or European regions [10]. Risk factors associated with mpox severity are varied, including HIV infection or other immunosuppressive conditions [11–15], pregnancy [16, 17], older [18, 19] or younger age [20, 21], viral clade [17], vaccination status [22], and comorbidity status [11]. While these risk factors are important in identifying and managing mpox cases, there is a lack of evidence on factors that influence the infectious period of mpox virus, particularly in an Asian context. In our scoping search, we found only one multicentre study among 541 Italian mpox patients examining this topic [23]. This study found that mucosal involvement and skin rash were predictors of prolonged mpox infection, and the duration of infectious periods and disease severity varied across racial groups [23].

**Figure 1.**
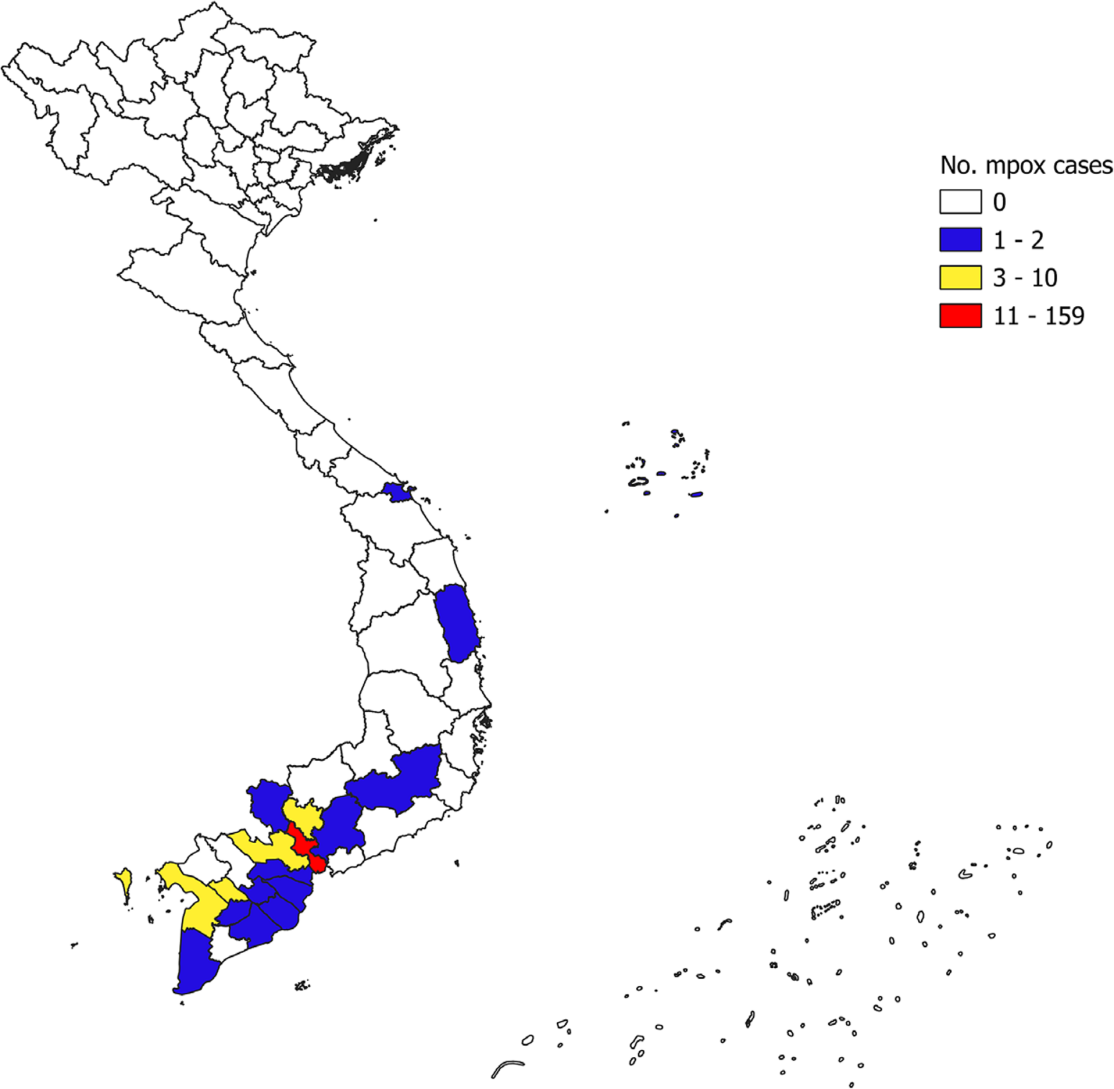
Distribution of mpox cases in the study as of 22 August 2024.

While mpox spread rapidly in Europe and the Americas, Asia remained largely unaffected until late 2022, with only limited cases reported [24]. However, in 2023, sustained transmission emerged in previously non-endemic Eastern and Southeast Asian countries [25]. In Vietnam, the first mpox case was detected on 23 September 2022. By August 2024, a total of 200 cases and eight fatalities had been reported nationwide, with most cases concentrated in the southern provinces [26] (Figure 1). Despite the increase in cases and its potential severity, there are concerns about the lack of empirical research evidence on mpox epidemiology in Asian countries. Many low- and middle-income countries in Asia, such as Vietnam, are struggling to provide adequate medical facilities, effective treatment, and vaccination for mpox. There is an urgent need to rapidly identify risk factors of the mpox infectious period to reduce the burden on their healthcare system and resources. Therefore, this study aimed to identify factors associated with the Mpox infectious period among the first 200 cases of Mpox in Vietnam using the Fine and Gray sub-distribution competing-risk regression model.

## Methods

### Case detection and definition

In Vietnam, a confirmed mpox case is identified through a positive polymerase chain reaction (PCR) test detecting the mpox virus. According to Vietnam Ministry of Health’s guidelines, the mpox cases are classified into three categories: asymptomatic cases, mild-to-moderate cases, and severe cases [6]. Asymptomatic mpox cases are patients without any clinical symptoms related to mpox. Moderate mpox cases are patients without comorbidities (other than mpox) who exhibit mild to moderate mpox clinical symptoms. These symptoms are diminished within 2 weeks without specific therapeutic interventions. Severe mpox cases are patients with pre-existing high risks of having severe mpox, such as pregnant women, older adults, children, those with comorbidity, those who are immunosuppressed. Patients with severe symptoms that persist for more than 2 weeks and requiring therapeutic interventions are also categorized as severe cases [6].

All mpox cases in Vietnam are tested for mpox every three or five days at designated healthcare facilities. Samples are collected from skin lesions using a swab. Patients are declared free of the mpox virus by a negative PCR test result (for asymptomatic cases) and the absence of active clinical symptoms, including no skin lesions for at least 48 h and the crusting of existing lesions (for mild to moderate and severe cases) [6]. During the infectious period, asymptomatic cases are required to isolate at home, and mild to moderate and severe cases must be isolated at designated healthcare facilities [6].

In this study, since the number of asymptomatic cases were too small for statistical analysis (n = 2), we categorized mpox patients by final treatment status instead, including (a) patients who received negative test for mpox virus, (b) patients who were deceased, or (c) patients whose status were lost-to-follow-up (LTFU). Lost-to-follow-up patients were those without a recorded date of receiving a negative test for mpox virus in our dataset.

### Variables

All data were extracted from Vietnam Mpox Management System.

#### Outcomes: Duration of mpox infectiousness

The duration of the mpox infectious period was defined as the time (in days) from the date of mpox symptom onset to the date of the first negative test result for the mpox virus. For asymptomatic patients, the infectious period was recorded from date of first positive test result for the mpox virus to date of first negative test result for the mpox virus.

#### Independent variables: Mpox clinical symptoms

Mpox clinical symptoms included: rash/mucosal lesions (on multiple body parts), fever, swollen lymph nodes, fatigue, itchiness, ulcer, sore throat, chill, severe headache, neck pain, cough, pink eyes, and nausea/vomit. These symptoms were recorded by healthcare professionals during the treatment period.

#### Independent variables: Health risk factors

From previous evidence presented above [11–21], we included the following health risk factors of the mpox infectious period: pregnancy status (Yes/No), mpox vaccination status (Yes/ No), comorbidity (other than mpox) (Yes/ No), HIV-diagnosed status (Yes/ No), syphilis-diagnosed status (Yes/ No), and number of treatment facilities for mpox (one facility/two or three facilities).

#### Other covariates

Sociodemographic covariates include age groups (18–29 years old/ 30–34 years old/ 35 or above), sex (Male/ Female), ethnicity (Kinh – the predominant ethnicity in Vietnam/Others), employment status (Full-time employed/Part-time employed/ Freelance/ Student), nationality (Vietnamese/Others), sexual orientation (men who have sex with men [MSM]/Others). Travel history covariates include travel during 21 days prior to first mpox symptom onset (Yes/ No), travel after first mpox symptom onset (Yes/ No), any animal contact during 21 days prior to first mpox symptom onset (Yes/ No).

### Statistical analysis

We first described mpox clinical symptoms, health risk factors, and other covariates, stratified by patients’ final treatment status (received negative test for mpox virus, deceased, or LTFU).

To assess factors associated with the mpox infectious period, we employed Fine and Gray sub-distribution competing-risk regression models on mpox symptoms and health risk factors. To investigate factors associated with a time-to-event outcome, survival analysis is commonly performed using the Kaplan – Meier method [27]. However, this method does not account for competing risk events that preclude the occurrence of the primary event, such as death. In such cases, Fine and Gray sub-distribution competing-risk regression model is used as a substitute. This model accounts for competing risks while evaluating the relative effects of covariates on time-to-event outcomes [28, 29].

We first plotted cumulative incidence functions (CIFs) to compare the crude incidence rates of cleared infection, stratified by mpox symptoms and health risk factors, and accounted for competing risks of death. The CIFs were derived from the univariable Fine and Gray model. Next, from the univariable model, each covariate was assigned to a log-likelihood value. Then, we built the multivariable model by adding covariates one by one into the model, starting from the one with the lowest log-likelihood to the one with the highest log-likelihood [29]. With each covariate added, we chose the most suitable model by comparing the goodness of fit using Akaike Information Criterion (AIC) and Bayesian Information Criterion (BIC). The final model included only covariates that reduced AIC or BIC upon addition [29] and reported sub-distribution hazard ratios (SHR) and 95% confidence intervals (CI). To account for the proportional hazard assumptions of the models, we tested for Schoenfeld residuals [29]. To ensure no collinearity among covariates, we used a correlation matrix and variance inflation factor (VIF) analysis, with a threshold of 10 for collinearity [30]. All VIF values are approximately one. All analyses were conducted using Stata software (version 18.0).

## Results

Among 200 mpox patients reported in Vietnam, 74% (148) received negative test for mpox virus, 4% (8) were deceased, and 22% (44) of patients were LTFU (Table 1). The majority of patients were younger than 35 (69%). Nearly all were males (98%, n = 196), of Kinh ethnicity (97%, n = 194), and of Vietnamese nationality (99%, n = 198), and nearly half were full-time workers (44.5%, n = 89). Furthermore, 76% of patients identified as MSM, and no patients were pregnant or vaccinated against mpox. Two-thirds of participants had comorbidities aside from mpox (67.5%, n = 135). Nearly 60% of patients (n = 114) tested positive for HIV, and 22.5% (n = 45) tested positive for syphilis. Most patients (73.5%, n = 147) were treated for mpox at one facility, while 26.5% (n = 53) received mpox treatment at two or three facilities. Regarding travel history, 19.5% (n = 39) travelled within 21 days prior to symptom onset, 8% (n = 16) travelled after symptom onset, and 2.5% (n = 5) reported animal contact within 21 days before onset.

**Table 1.** Sociodemographic of mpox patients, stratified by final treatment status (N = 200)

Factors	Total (n = 200)	Final treatment status, n (%)			*p*-value
		Negative for Mpox virus (n = 148, 74%)	Lost to follow-up (n = 44, 22%)	Deceased (n = 8, 4%)	
**Sociodemographic covariates**					
**Age group**					0.65
18–29	72 (36.0)	52 (35.1)	18 (40.9)	2 (25.0)	
30–34	66 (33.0)	51 (34.5)	11 (25.0)	4 (50.0)	
35+	62 (31.0)	45 (30.4)	15 (34.1)	2 (25.0)	
**Sex**					<0.001
Male	196 (98.0)	148 (100.0)	40 (90.9)	8 (100.0)	
Female	4 (2.0)	0 (0.0)	4 (9.1)	0 (0.0)	
**Ethnicity**					0.23
Kinh	194 (97.0)	145 (98.0)	41 (93.2)	8 (100.0)	
Others	6 (3.0)	3 (2.0)	3 (6.8)	0 (0.0)	
**Employment status**					<0.001
Fulltime	89 (44.5)	76 (51.4)	8 (18.2)	5 (62.5)	
Parttime	53 (26.5)	28 (18.9)	23 (52.3)	2 (25.0)	
Freelancer	48 (24.0)	35 (23.6)	12 (27.3)	1 (12.5)	
Student	10 (5.0)	9 (6.1)	1 (2.3)	0 (0.0)	
**Nationality**					0.70
Vietnamese	198 (99.0)	146 (98.6)	44 (100.0)	8 (100.0)	
Others	2 (1.0)	2 (1.4)	0 (0.0)	0 (0.0)	
**Sexual Behavior**					0.52
MSM	152 (76.0)	115 (77.7)	32 (72.7)	5 (62.5)	
Others	48 (24.0)	33 (22.3)	12 (27.3)	3 (37.5)	
**HEALTH RISK FACTORS**					
**Pregnancy status**					–
No	200 (100.0)	148 (100.0)	44 (100.0)	8 (100.0)	
**Mpox vaccination status**					–
No	200 (100.0)	148 (100.0)	44 (100.0)	8 (100.0)	
**Comorbidity other than Mpox**					0.100
No	65 (32.5)	48 (32.4)	17 (38.6)	0 (0.0)	
Yes	135 (67.5)	100 (67.6)	27 (61.4)	8 (100.0)	
**HIV-diagnosed status**					0.043
Negative	86 (43.0)	66 (44.6)	20 (45.5)	0 (0.0)	
Positive	114 (57.0)	82 (55.4)	24 (54.5)	8 (100.0)	
**Syphilis-diagnosed status**					0.096
Negative	155 (77.5)	111 (75.0)	39 (88.6)	5 (62.5)	
Positive	45 (22.5)	37 (25.0)	5 (11.4)	3 (37.5)	
**Number of treatment facilities for Mpox**					0.004
One facility	147 (73.5)	114 (77.0)	31 (70.5)	2 (25.0)	
Two or three facilities	53 (26.5)	34 (23.0)	13 (29.5)	6 (75.0)	
**Travel history**					
**Travel during 21 days prior to symptom onset**					0.32
No	161 (80.5)	122 (82.4)	32 (72.7)	7 (87.5)	
Yes	39 (19.5)	26 (17.6)	12 (27.3)	1 (12.5)	
**Travel after symptom onset**					0.24
No	184 (92.0)	139 (93.9)	38 (86.4)	7 (87.5)	
Yes	16 (8.0)	9 (6.1)	6 (13.6)	1 (12.5)	
**Any contact with animals during 21 days prior to symptom onset**					0.41
No	195 (97.5)	143 (96.6)	44 (100.0)	8 (100.0)	
Yes	5 (2.5)	5 (3.4)	0 (0.0)	0 (0.0)	

*Note:* p-value were calculated using Pearson’s chi-squared test.

Abbreviations: MSM, Men who have sex with men.

Among three groups of patients (negative for mpox virus, deceased, and LTFU), there were significant differences in terms of sex, employment status, HIV-positive mpox, and number of treatment facilities for mpox. All patients who tested negative for mpox virus and all deceased patients were males, while 9.1% (n = 4) of LTFU patients were females. More than half of the patients who tested negative for mpox virus (51.4%, n = 76) and deceased (62.5%, n = 5) patients were full-time workers, whereas 52.3% (n = 23) of LTFU patients worked part-time. All deceased patients were HIV-positive, while these figures were 54.4% (n = 24) among LTFU patients and 55.4% (n = 82) among patients who tested negative for mpox virus. Three-quarter (75%, n = 6) of deceased patients were treated for mpox at two or three facilities, compared to 29.5% of LTFU patients and 23% of patients who tested negative for mpox virus.

Table 2 reports mpox clinical symptoms stratified by final treatment status. Mpox patients exhibited a median of three symptoms (interquartile range [IQR]: 2–5) throughout the infectious period. The median symptom count was significantly higher among patients who tested negative for mpox virus (4, IQR: 2–5) and deceased patients (5, IQR: 3.5–6) than in patients who were LTFU (2, IQR: 1–2). Nearly all patients had at least one (98.5%, n = 198) or two symptoms (84.5%, n = 169), and 16% (n = 32) patients reported with six clinical symptoms. For all symptom counts, deceased patients had a significantly higher number of symptoms compared to those who tested negative for mpox virus and those LTFU.

**Table 2.** The clinical symptoms of mpox patients by event status (n = 200)

Factors	Total (n = 200)	Final treatment status, n (%)			p-value
		Negative for Mpox virus (n = 148, 74%)	Lost to follow-up (n = 44, 22%)	Deceased (n = 8, 4%)	
**Number of symptoms (median, IQR)**	3.0 (2.0, 5.0)	4.0 (2.0, 5.0)	2.0 (1.0, 2.0)	5.0 (3.5, 6.0)	<0.001
**Presence of symptoms**					
One symptom	198 (98.5)	148 (100.0)	41 (93.2)	8 (100.0)	0.005
Two symptoms	169 (84.5)	134 (90.5)	27 (61.4)	8 (100.0)	<0.001
Three symptoms	119 (59.5)	110 (74.3)	1 (2.3)	8 (100.0)	<0.001
Four symptoms	84 (42.0)	78 (52.7)	–	6 (75.0)	<0.001
Five symptoms	56 (28.0)	51 (34.5)	–	5 (62.5)	<0.001
Six symptoms	32 (16.0)	29 (19.6)	–	3 (37.5)	0.002
**Clinical symptoms**					
Rash/mucosal lesions	197 (98.5)	148 (100.0)	41 (93.2)	8 (100.0)	0.005
Rash/mucosal lesions on multiple body parts	102 (67.5)	96 (66.2)	25 (56.8)	6 (100.0)	0.083
Fever	132 (66.0)	99 (66.9)	–	8 (100.0)	0.054
Swollen lymph nodes	60 (39.7)	54 (37.2)	–	6 (100.0)	0.002
Fatigue	57 (37.7)	55 (37.9)	–	2 (33.3)	0.82
Itchiness	57 (37.7)	56 (38.6)	–	1 (16.7)	0.28
Ulcer	56 (28.0)	45 (30.4)	3 (6.8)	8 (100.0)	<0.001
Sore throat	33 (21.9)	32 (22.1)	–	1 (16.7)	0.75
Chill	30 (19.9)	29 (20.0)	–	1 (16.7)	0.84
Severe headache	29 (19.2)	28 (19.3)	–	1 (16.7)	0.87
Neck pain	25 (16.6)	25 (17.2)	–	0 (0.0)	0.27
Cough	17 (11.3)	15 (10.3)	–	2 (33.3)	0.081
Pink eye	7 (4.6)	7 (4.8)	–	0 (0.0)	0.58
Nausea/vomit	5 (3.3)	4 (2.8)	–	1 (16.7)	0.062

*Note:* p-value were calculated using Pearson’s chi-squared test and Kruskal-Wallis – no data recorded.

The most common clinical symptoms reported were rash/mucosal lesions (98.5%, n = 197), followed by rash/ mucosal lesions on multiple body parts (67.5%, n = 102). Fever was reported by 66% (n = 132) patients, and lymph nodes by 39.7% (n = 60) patients. Fatigue and itchiness were each reported among 37.7% (n = 57) patients, followed by ulcers (28.0%, n = 56), sore throat (21.9%, n = 33), chills (19.9%, n = 30), severe headache (19.2%, n = 29), neck pain (16.6%, n = 25), and cough (11.3%, n = 17). The least commonly reported symptoms are pink eye (4.6%, n = 7) and nausea/vomiting (3.3%, n = 5). There were significant differences in the number of deceased patients and those who tested negative for the mpox virus in reporting symptoms of rash/mucosal lesions on multiple body areas, fever, lymph nodes, and ulcers. All deceased patients had multiple body rashes/mucosal lesions, fever, lymphadenopathy, and ulcers, compared to about 30% of patients who tested negative for mpox virus reporting such symptoms.

**Figure 2.**
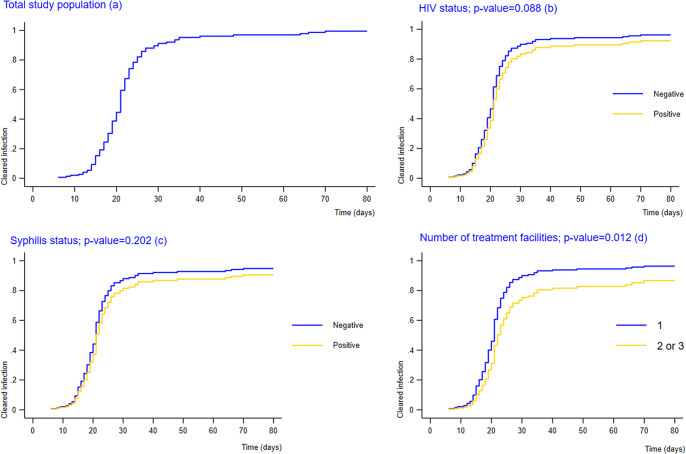
Cumulative Incidence Functions of duration of mpox infectiousness among 200 mpox patients, stratified by (a) Total Study Population, (b) HIV positive status, (c) Syphilis positive status, and (d) Number of mpox treatment facilities.* The cumulative incidence function was estimated using Fine and Gray method and was interpreted as incidence of cleared infection. P-value was calculated from Gray’s test.** mpox infectiousness was defined as the time from the date of symptom onset or first positive test to the date of the first negative test result for the mpox virus.

Figure 2 shows the CIFs for mpox infectious periods, accounted for decease status (Panel A). The CIFs were stratified by three health risk factors: HIV-positive status (Panel B), syphilis-positive status (Panel C), and number of treatment facilities for mpox (panel D). We did not include other health risk factors, such as pregnancy status or vaccination since the recorded data was too small for meaningful regression. In all four panels, the cumulative incidence curve rises steeply between 0 and 30 days before reaching a plateau. This indicates that most of the mpox infectious periods were recorded within the first 30-day period. Specifically, in panel A, the rate of cleared infection was 2% after the first 10 days from symptom onset, increasing to 15.5% after 15 days, 46% after 20 days, 85% after 25 days, and 93% after 30 days. Panel B and C show that mpox patients who were HIV-positive or syphilis-positive had a lower incidence rate of cleared infection (i.e. had longer mpox infectious periods) than those who were negative for HIV and syphilis, although the differences were not statistically significant. Panel D shows that mpox patients who were treated for mpox at two or three facilities had a significantly lower incidence rate of cleared infection (i.e. had significantly longer mpox infectious periods) compared to those who were treated at a single facility (p-value = 0.012).

**Figure 3.**
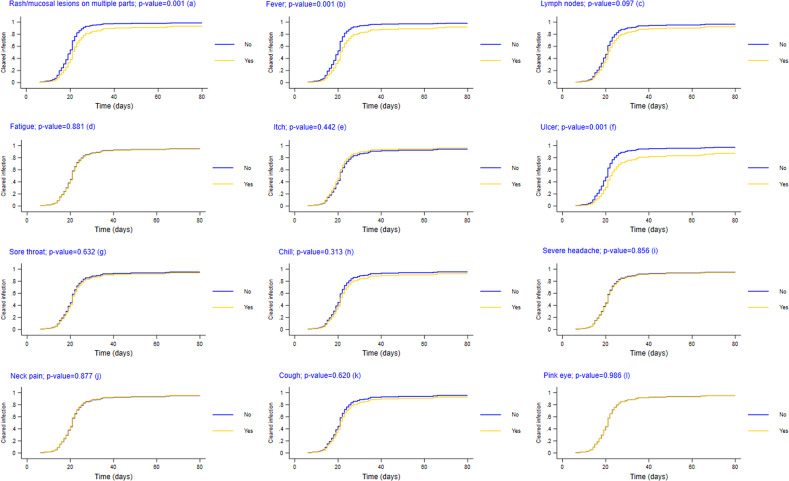
Cumulative Incidence Functions of duration of mpox infectiousness among 200 Mpox patients by clinical symptoms onset.* The cumulative incidence function was estimated using Fine and Gray method and was interpreted as incidence of cleared infection. P-value was calculated from Gray’s test.** mpox infectiousness was defined as the time from the date of symptom onset or first positive test to the date of the first negative test result for the mpox virus.

Figure 3 presents the CIFs for mpox infectious periods, stratified by their clinical symptoms. Patients who reported symptoms of rash/mucosal lesions on multiple body parts, fever, lymph nodes, or ulcers had longer infectious periods than patients without such symptoms. Specifically, by day 30, the rate of cleared infection was 86.7% among patients reporting rash/mucosal lesions compared to 98.0% in patients without such symptoms. Ninety-eight percent of patients who did not report fever tested negative for mpox infection within 30 days, while this figure was 85.0% for those who were feverish. The rate of cleared infection was 92.3% and 93.2% in patients without lymph nodes and ulcers by day 30, compared to 87.5% and 81.5% in those with symptoms in lymph nodes and ulcers, respectively.

Table 3 shows the univariable and multivariable Fine and Gray regression models for mpox infectious periods. Symptoms of rash/mucosal lesions on multiple body parts, fever, lymph nodes, and ulcers are selected covariates that met the inclusion criteria for goodness of fit models and were retained in the multivariable model. The probability of ending infectious periods was lower among mpox patients who reported rash/mucosal lesions on multiple body parts, fever, lymphadenopathy, or ulcers. Specifically, patients with rash or mucosal lesions had a significantly lower SHR (0.62, 95% CI: 0.46–0.83, p = 0.001) in the univariable model, indicating a longer time of mpox infectious periods than those without these symptoms. This result remains significant in the multivariable model (SHR = 0.71, 95% CI: 0.51–0.99, p = 0.041). In both the univariable and multivariable models, patients with lymph nodes had a lower SHR, although these findings were not statistically significant. The presence of ulcers was associated with a significantly lower SHR compared to the absence of such symptoms in the univariable model (0.57, 95% CI: 0.41–0.80, p = 0.001), suggesting a longer time of mpox infectious periods. This association remained significant in the multivariable model (SHR = 0.65, 95% CI: 0.47–0.90, p = 0.010). The univariable model showed a significantly lower SHR among patients reporting fever compared to patients not reporting fever (0.62, 95% CI: 0.46–0.83, p = 0.001). However, this effect was no longer statistically significant in the multivariable model.

**Table 3.** Univariable and multivariable Fine-Gray competing-risk regression models for duration of mpox infection among 200 mpox Patients

Factors	Univariate model			Multiple model			
	SHR	95%CI	p-value	SHR	95%CI	p-value
**Rash/mucosal lesions on multiple body parts**						
No	Ref			Ref		
Yes	0.62	0.46, 0.83	0.001	0.71	0.51, 0.99	0.041
**Lymph nodes**						
No	Ref			Ref		
Yes	0.76	0.55, 1.05	0.097	0.82	0.58, 1.16	0.265
**Ulcer**							
No	Ref			Ref		
Yes	0.57	0.41, 0.80	0.001	0.65	0.47, 0.90	0.010
**Fever**							
No	Ref			Ref		
Yes	0.62	0.46, 0.83	0.001	0.73	0.52, 1.05	0.087

Abbreviations: SHR, sub-hazard ratio; CI, confidence intervals.

## Discussion

This study represents a pioneering effort to explore risk factors associated with the mpox infectious period among the first 200 mpox cases in Vietnam. Key findings reveal that most mpox cases recovered within 30 days. HIV-positive status, mpox treatment in multiple health facilities, and the presence of mpox-like symptoms, particularly mucosal lesions, ulcers, and fever, were identified as predictors of prolonged mpox infectious periods. These results emphasize the need for targeted risk stratification, treatment, and isolation frameworks for mpox case management and control.

Our findings reveal that nearly all mpox patients experienced infectious periods of 30 days or less. While existing evidence, predominantly from European settings, showed that mpox viral clearance from biological fluids predominantly occurs within four weeks from disease onset [31, 32], there are a few differences in reported infectious periods of mpox. Previous studies from Italy and Spain reported longer infectious periods of up to 33 days and 41 days, respectively [31, 32], while one study in France demonstrated relatively faster viral clearance, with most samples testing mpox virus-negative or weakly positive within 14 days after symptom onset [33]. The discrepancy warrants more in-depth epidemiological studies to compare international data of mpox cases.

Our results also showed that mpox patients who reported multiple mpox-like symptoms, such as rash, mucosal lesions, ulcers, and fever, are at risk of prolonged infectious period and should be prioritized for isolation and close monitoring. Rash and mucosal lesions are well-established risk factors for mpox severity and infectiousness, as shown in the recently developed Mpox Severity Scoring System (MPOX-SSS) [34]. However, ulcers and fever are less common than mucosal lesions in current literature [35, 36]. Our findings emphasize that these symptoms are critical indicators of prolonged infectiousness and should be considered to identify high-risk mpox patients. This evidence further underscores the importance of symptom-based management protocols for mpox to reduce transmission risks and enhance patient outcomes.

Our findings revealed that HIV-positive mpox patients had significantly longer infectious periods compared to HIV-negative patients, which is consistent with existing literature [23]. This may be attributed to the immunosuppression condition caused by HIV, which impairs the host’s ability to effectively clear the mpox virus [37]. While further investigation into the effect of HIV on mpox infectious periods is needed, it is notable that all mpox fatalities in our dataset were HIV-positive individuals. A higher risk of mpox mortality was also found in a previous study in Nigeria, which reported a 14-time higher mortality risk of mpox in persons living with HIV compared to those without HIV coinfection [14]. Individuals with HIV account for approximately 40–50% of mpox cases [38, 39], and severe and fatal outcomes are more common among mpox patients with advanced or uncontrolled HIV infection [37]. Therefore, we underscore the need for mpox surveillance and vaccination policies targeted at this vulnerable population.

In our findings, receiving mpox treatment across multiple healthcare facilities is associated with prolonged mpox infectious periods. This may be explained by the newly developed mpox treatment strategy in Vietnam, which remains fragmented across healthcare facilities nationwide. The absence of a consistent case management strategy may lead to delay in accessing essential diagnostic tests and treatments for mpox cases identified in lower-tier healthcare systems. Patients may face extended waiting periods before being transferred to appropriate healthcare facilities, often without adequate isolation or treatment during their infection periods. This practice may increase the risk of mpox exposure to other patients or related healthcare professionals [40]. Given the recent emergence of mpox infections in Vietnam and other Asian countries, our findings highlight an urgent need for a better coordinated mpox case management system. Such system should prioritize accessible testing and treatment facility, alongside a nationwide proactive strategy for mpox surveillance.

### Limitations and strengths

We acknowledge several limitations in our study. First, we could not include data on mpox symptom duration, as it was not collected in the Vietnam Mpox Management System. Second, while the current Vietnamese guidelines define the cleared infection as a combination of a negative mpox virus test and symptom resolution, data on the exact date of symptom clearance were unavailable. As a result, we used the date of a negative test as a proxy for the end of infection. These data could offer valuable insights into the link between symptom persistence and infectious periods. Third, we did not assess the potential influence of mpox viral clades in this study. While different clades may have distinct clinical and epidemiological characteristics, the predominant clades circulating in Vietnam during the study period were Clade IIb [26]. Therefore, we expected minimal variation in clinical or epidemiological characteristics among our cases. Fourth, although presymptomatic transmission has been documented in mpox cases [41, 42], our dataset lacks information on incubation time and presymptomatic transmission. Fifth, data on the serial interval and incubation period were not available in our dataset, which limited our ability to assess the dynamics of disease transmission and the timing of symptom onset relative to exposure. Sixth, our dataset was insufficient for further analysis of well-established mpox risk factors, such as pregnancy, sexual orientation, or vaccination status, as it included only the first few mpox cases in Vietnam. We recommend that future studies incorporate detailed symptom timelines and clinical data to provide a more comprehensive understanding of the relationship between symptom resolution and infection duration.

Despite these limitations, this study has several notable strengths. First, our analysis represents national mpox case data, providing a comprehensive overview of mpox epidemiology in Vietnam and similar settings. Additionally, the use of a competing-risk model enabled advanced insights into factors associated with prolonged Mpox infectious periods while accounting for the competing risk of death. Our findings also underscore the vulnerability associated with prolonged infectious periods among HIV-positive mpox patients and highlight the challenges posed by fragmented mpox case management systems in low- and middle-income countries. As mpox continues to be a global public health concern, our findings should guide the development of effective mpox case management strategies, particularly in Asian countries with sporadic cases reported. These strategies should include comprehensive risk stratification for vulnerable populations and emphasize coordinated efforts in surveillance, treatment, and isolation of mpox patients.

## Conclusions

This study identified risk factors for prolonged mpox infectious period using national data from Vietnam. HIV-positive status, receiving treatment in multiple health facilities, and the presence of multiple mpox-like symptoms – particularly mucosal lesions, ulcers, and fever – were associated with prolonged mpox infectious periods. Our findings are valuable for the development of effective mpox case management and treatment in anticipation of future mpox epidemic concerns.

## Data Availability

Raw data supporting the findings of this study are available from the corresponding author upon reasonable request.
